# Human‐specific α7nAChR‐dependent adaptation to mechanical properties of the extracellular environment

**DOI:** 10.1002/alz.095019

**Published:** 2025-01-09

**Authors:** Anna Marie Szombathy, Ivanna Ihnatovych, Ryu Dorn, Erik Nimmer, Yuna Heo, Yongho Bae, Kinga Szigeti

**Affiliations:** ^1^ University at Buffalo, Buffalo, NY USA

## Abstract

**Background:**

*CHRFAM7A* is a human‐restricted gene associated with neuropsychiatric and neurodegenerative disorders. The translated CHRFAM7A protein incorporates into the α7 nicotinic acetylcholine receptor (α7nAChR) leading to a hypomorphic receptor. Mechanistic insight from isogenic iPSC derived neuronal and mononuclear cells demonstrated that *CHRFAM7A* affects Ca^2+^ signaling and activates small GTPase Rac1 leading to an actin cytoskeleton gain of function. Fundamental differences in tissue stiffness in human and rodent brain are emerging as a translational gap. Since the actin cytoskeleton is a driver of adaptation to tissue stiffness, we explored the role of CHRFAM7A in mechanobiology.

**Method:**

Neuronal progenitors and monocytes were differentiated from isogenic iPSCs (null and CHRFAM7A knock‐in (KI)). Polyacrylamide hydrogels corresponding to Young’s modulus 2 kPa (soft) and 5 kPa (stiff) were used to model the mechanical properties of the tissue environment. Actin immunofluorescent morphological analysis and atomic force microscopy (AFM) were utilized to characterize cellular adaptation.

**Result:**

AFM demonstrated intracellular stiffness adaptation to the soft and stiff environment in CHRFAM7A KI neuronal progenitors and monocytes. In CHRFAM7A KI neuronal progenitors, growth cone (GC) morphology adapted to the stiffness by reinforcing lamellipodia and maintaining directionality. In null MGE progenitors filopodia remained the actin structure in both the soft and stiff environment resulting in GC curvature in the stiff hydrogel. Polarization pattern favored the bipolar morphology in both the neuronal and monocytic lineage of CHRFAM7A KI cells in the stiff environment, while null cells retained the multipolar pattern. Cell polarity correlated with small GTPase activity: in null MGE progenitors, multipolar morphology is associated with high RhoA activity in the stiff environment. In contrast, CHRFAM7A KI MGE progenitors adapted to stiffness by activating both CDC42 and Rac1.

**Conclusion:**

Human specific *CHRFAM7A* facilitates adaptation to matrix stiffness in both the neuronal and mononuclear lineage, bridging the gap between rodent and human brain stiffness and holding implications for physiological and pathological conditions. Human‐restricted *CHRFAM7A* needs to be taken into account to overcome the translational gap in drug development for neurodegenerative and neuroinflammatory diseases.

